# Female Germ Cell Development, Functioning and Associated Adversities under Unfavorable Circumstances

**DOI:** 10.3390/ijms22041979

**Published:** 2021-02-17

**Authors:** Dinesh Bharti, Manisha Tikka, Sang-Yun Lee, Eun-Yeong Bok, Hyeon-Jeong Lee, Gyu-Jin Rho

**Affiliations:** 1 Department of Theriogenology and Biotechnology, College of Veterinary Medicine and Research Institute of Life Sciences, Gyeongsang National University, Jinju 52828, Korea; bhartidinesh54@gnu.ac.kr (D.B.); sy_lee@gnu.ac.kr (S.-Y.L.); eybok@gnu.ac.kr (E.-Y.B.); 2 Department of Zoology and Environmental Sciences, Punjabi University, Patiala 147002, India; manisha_rs@pbi.ac.in; 3 Department of Medicine, University of California, San Diego, CA 92093-0021, USA; hjlee@health.ucsd.edu

**Keywords:** gametogenesis, oocytes, pathways, signaling, stem cells, differentiation, tumor, cancer

## Abstract

In the present era, infertility is one of the major issues which restricts many couples to have their own children. Infertility is the inability to achieve a clinical pregnancy after regular unprotected sexual intercourse for the period of one year or more. Various factors including defective male or female germ cell development, unhealthy and improper lifestyles, diseases like cancer and associated chemo-or-radiation therapies, congenital disorders, etc., may be responsible for infertility. Therefore, it is highly important to understand the basic concepts of germ cell development including primordial germ cell (PGC) formation, specification, migration, entry to genital ridges and their molecular mechanisms, activated pathways, paracrine and autocrine signaling, along with possible alteration which can hamper germ cell development and can cause adversities like cancer progression and infertility. Knowing all these aspects in a proper way can be very much helpful in improving our understanding about gametogenesis and finding possible ways to cure related disorders. Here in this review, various aspects of gametogenesis especially female gametes and relevant factors causing functional impairment have been thoroughly discussed.

## 1. Introduction

Ability to produce the whole new organism and transfer of valuable genetic and epigenetic information from one generation to another, makes germ cells a very unique and most valuable special type of cells. Germ cell development starts from a very small number of precursor cells called primordial germ cells (PGCs) at around E5.5–6.5 in mice, and the third–fourth week of gestation in humans [1,2]. Going through various signal cascades from the surrounding neighboring cells, PGCs gradually repress their somatic gene activity while attaining germ cell specific signature [3]. For the formation of healthy gametes, PGCs have to go through epigenetic modifications [1]. During whole course, any deformity or issues related with the expression or repression of stage specific genes produce developmental defects, impaired functioning, and sometimes incapability of the produce (gametes) to fertilize, i.e., infertility. Such unwanted changes may result into germ cell-specific tumors (GCTs). One possible therapy for such complications can be the use of stem cells as therapeutic agents as they can be helpful in deriving germ-like cells in vitro and efficiently transplanted to eradicate fertility associated complications. In our previous review of literature [4], we have thoroughly discussed about how oocyte-like cells can be generated in vitro with the help of stem cells. These stem cell-based in vitro differentiated gamete models can also provide an assistance in knowing basic mechanisms to greater extent. Therefore, knowing exact mechanisms related to germ cell development and possible adversities which can cause impaired functioning may be highly valuable to overcome related issues and also proved extremely helpful to develop relevant medicines. Selection of healthy gametes makes the basis of future healthy generations. Therefore, with the help of gametes specific valuable understandings, a healthy world free of future diseases can be made possible. Moreover, endangered species can also be preserved either by generating multiple copies or by restoring their gametes or associated tissues so that with the help of scientific advancements they can be restored for the future generations.

The main purpose of this review is to describe about various aspects of gamete development including development route, signaling pathways, time course expression and repression of various genes, along with associated in utero and postnatal complications and their after-results in the form of tumors or functional impairment. We have mainly focused on female gamete development and concerned complications and tried to discuss these mechanisms in mouse and humans.

## 2. Female Germ Cell: In Vivo Developmental Stages (Primordial Germ Cells to Oocyte)

Before attaining full maturity, female germ cells go through various developmental stages. Brief description about these stages is as follows:

### 2.1. Primordial Germ Cell Development

**Figure 1:**
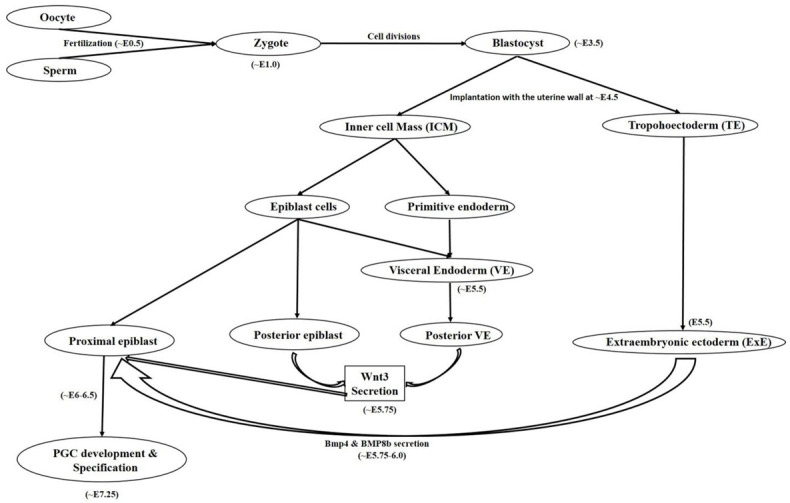
Diagrammatical outflow of the PGC development and specification in mice. Oocyte and sperm after getting fertilized produces zygote. While undergoing further cell divisions, zygote attain blastocyst stage at around embryonic development day 3.5 (~E3.5) having an outer trophoblast cell monolayer and pluripotent inner cell mass (ICM) which is further separated into epiblast and primitive endoderm. Implantation of the blastocyst with the uterine wall takes place at ~E4.5. Further developmental stages are comprised of extraembryonic ectoderm (ExE) development from trophoectoderm and visceral endoderm (VE) from primitive endoderm. Furthermore, Wnt3 is secreted by both of the posterior epiblast and VE and BMP4 & BMP8b secreted by ExE. Wnt3 and BMPs secretions finally assists in production of a few primordial germ cells (PGCs) from proximal epiblast and finally PGC specification takes place.

Mammalian germ cell development begins in the form of PGCs which are the embryonic precursors of gametes [1,2] which on later stages becomes male or female gametes often termed as sperm and oocyte, respectively. Here in this review we will mainly discuss both the human and mouse female germ cell development in vivo and in vitro. A number of developmental changes initiated from proximal epiblast cells, later accompanied by attainment of various markers expression under different pathway activations are held responsible for developing a mature sperm or oocyte with functional ability. The developmental route mainly comprised of following steps: gastrulation → epiblast cells formation → primordial germ cell → gonocyte → oogonium → primary oocyte → secondary oocyte → antral follicle → mature oocyte. Earlier developmental processes are under the control of both of the WNT3 and bone morphogenic protein (BMP) family transcription factors and activation of pathways associated with them. In the mouse at around E5.75, PGCs formation is governed by WNT3 secretion by posterior visceral endoderm (VE) and posterior epiblast cells which is further followed by secretion of BMP family member proteins such as BMP4 and BMP8b by extraembryonic ectoderm (ExE) at around E6.0 [1]. WNT3 signaling regulates PGC induction by its downstream target Brachyury, a mesodermal marker which promotes the expression of both somatic as well as germ cell markers, whereas BMP4 governs the suppression of somatic genes while up regulating germline determinant genes *Blimp1* and *Prdm14* [5]. *BMP8b* also plays important role in mouse PGC generation. In a mouse study, null mutations in *BMP8b* were shown to cause complete lack of PGCs whereas reduced number of PGCs were observed in *BMP8b* heterozygotes [6]. Many other reports have also shown severe reduction in the number of PGCs from mice embryos in which targeted deletion of either *BMP2*, *BMP4*, and *BMP8b* or their downstream signaling components including *Smad1*, *4*, *5* was performed [7,8,9,10,11]. Similarly, no PRDM1 positive PGCs were observed in mutant mouse embryos which undergo targeted deletion of *Wnt3* [5]. The Wnt signaling pathway involving Wnt5a and its receptor Ror2 has also been associated with successful migration and functionality of PGCs [12]. These findings strongly advocate the utility and role of BMP and Wnt signaling pathways in development, migration and proper functioning of the developing PGCs which further determines the specification and development of gametes. It is worthy to notice the indispensable role of *Fragilis*, *Smad1*, and *Stella* which helps in the specification of PGCs and also provides distinction from somatic cells [13,14,15]. Any deformity or suppression of these valuable factors results into impaired PGC/gamete production and hampers their functional ability. Beginning with a small number of ~30–40 cells, PGCs proliferate and finally migrates towards genital ridges where they undergo genome wide DNA demethylation, histone modification, imprinting erasure and reactivation of X chromosome [16,17]. Until this stage, sex specification is not achieved as PGCs are bipotent in nature and express common germ cell markers shared by both male and female gametes. In humans, PGC specification begins at around the third gestational week during which erasure of DNA methylation takes place [18,19,20,21]. Differences have been observed among transcription factors requirement for PGCs specification and maintenance among humans and mice [22,23,24]. During progressive stages, cells undergo a transitional shift from pluripotency associated marker expression to germ cell specificity in a gradual manner and attain expression of early PGC makers, late PGC markers, meiosis markers, undergo epigenetic reprograming and finally attains mature germ cell markers [19]. While analyzing the transcriptome of human PGCs, Guo and colleagues [19] found that both pluripotency and germ cell specific genes are simultaneously expressed from their migrating to the gonadal stage. Diagrammatical outflow of the PGC development and specification has been given in Figure 1.

### 2.2. Primordial Germ Cell Migration

**Figure 2:**
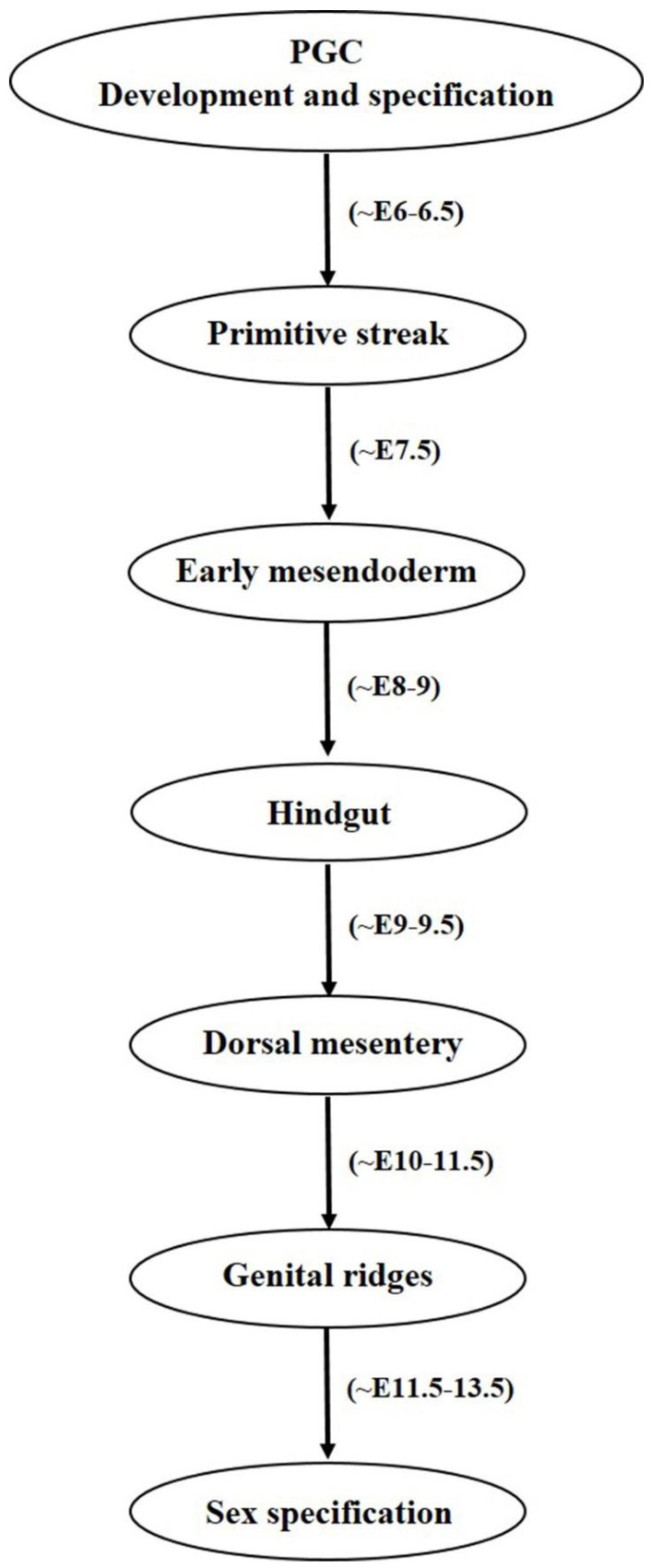
Diagrammatical outflow of the PGC migration. PGCs undergo a series of events before reaching gonadal ridges and finally sex specification. They are identified as a few precursor cells at the base of allantois and specified under the effect of molecular cues associated with Wnt3 and BMP pathways. After passing through a number of stages, PGCs finally enter genital ridges and specified as male and female gametes.

Once the PGCs are formed, their migration to gonadal ridges constitutes an important developmental step towards gamete specification and their proper functioning. It is worth noticing that the concept of signaling guidance for PGCs towards the gonads was first of all described in zebrafish [25]. Both intrinsic motility factors and external guidance cues are required by the PGCs to successfully migrate and maintain their survivability [26]. In the beginning, BMP signaling from ExE governs PGC formation from epiblast cells, which are further mediated by *BLIMP1* or *PRDM1* transcriptional regulators which not only stimulate PGC specific gene such as *Stella* but also suppresses the expression of somatic cell genes cascade [27,28]. Like *BLIMP1*, *PRDM14* also regulates mouse PGC specification, lacking or knockdown of both the factors either resulted into improper PGC differentiation and defected migration or induced sterility in mice models [29,30]. Along with *BLIMP1* and *PRDM14*, *AP2γ* has also been found essential in regulating mammalian PGC specification [31]. *AP2γ* is expressed by mouse PGCs from E7.25 to E12.5 and its targeted disruption has resulted into male and female sterility. It was also believed that PGC migration in mouse is also coordinated by interferon induced transmembrane protein 1 (*IFITM1*), as RNA interference induced *IFITM1* knockdown in the primitive streak resulted into PGC migration failure towards endoderm, indicating possible role of *IFITM1* in PGC migration from mesoderm to endoderm [32]. However, contradicting results were shown by Lange UC et al. (2008), where embryonal deletion of the *IFITM1* gene family could not cause impaired PGC migration [33]. During the period of migration and arrival of PGCs towards hindgut, *Nanos3* plays multiple vital roles by maintaining germ cell’s undifferentiated state, protecting PGCs against apoptosis by suppressing Bax-dependent and independent pathways and thereby helps in germ cell development [34]. *Nanos3* is expressed by migrating PGCs at various germ cell specific developmental stages in both testis and ovaries. Moreover, without its expression, germ cell detection in the ovary and testis is not possible [15,34]. Failure in migration can cause developmental issues and infertility, and also may cause teratoma formation in extra gonadal regions [12]. At around E11.5, most PGCs are migrated towards genital ridges and become populated there at around E12.5. During later stages, establishment of sexually dimorphic sex cords takes place between E13.5–17.5 and birth and most of the ectopic PGCs die due to apoptotic pathways and rest of them transforms into male and female gametes in their respective sex gonads [15]. Researchers believed that PGCs lose their migration properties once they associate with somatic cells and reach gonads [26]. One possible reason for their ceasing behavior may be due to the cell-to-cell interactions between PGCs and somatic cells. Proper mechanism behind this stoppage is not known and need in depth elucidation. Diagrammatical outflow of the PGC migration has been given in Figure 2.

## 3. Functional Efficacy of the Female Germ Cells

Ability to generate offspring by either getting fertilized with healthy spermatocyte or parthenogenetically has been the prime function of the oocytes. Unlike male gametes, female germ cells (oocytes) have been thought to have limited number which decline with the age and there has been contradictory views regarding regenerative activity in juvenile or adult ovaries. Pathological conditions such as cancer or ovarian disorders including premature ovarian failure (POF) and premature ovarian insufficiency (POI) put additional threats to female reproductive life and ultimately results into infertility. Therefore, researchers throughout the globe have consistently made extensive efforts to develop methodologies so that functional efficacy of the in vitro derived oocytes can be enhanced. Although, due to complexities in deriving oocyte-like cells (OLCs) in vitro (ranging from ethical issues concerned with use of pluripotent ESCs or iPSCs, low response by less potent MSCs, or xenogeneic concerns regarding use of follicular fluid or specialized cells from different organisms), not much success has been achieved. Only a few researchers have reported derivations of healthy offspring by using ovary-derived cells, i.e., premeiotic fetal germ cells, germline stem cells, primordial germ cells, granulosa cells, and ovarian stem cells especially in nonhuman species (murine) [35,36,37,38,39]. However, getting the in vitro derived fertilizable oocytes and finally healthy progeny is still an unachieved distant milestone. Similarly, in vitro differentiation into fertilizable OLCs using adult MSC sources other than ovary derived cells is still far away. Therefore, many efforts will be needed to achieve this goal.

## 4. Developmental Impairments and Associated Abnormalities

**Figure 3:**
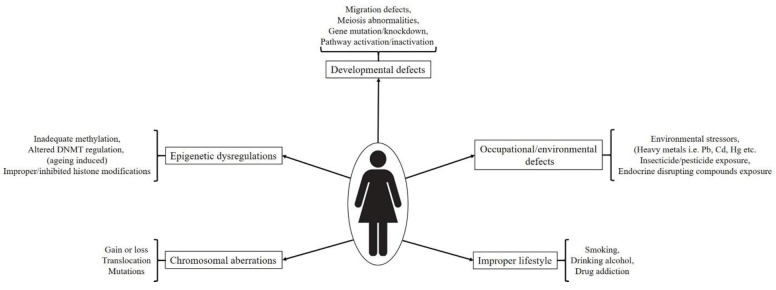
Diagrammatical representation of factors affecting germ cell development and their functionality. All germ cell developmental stages are affected by a number of factors including mutations, chromosomal abnormalities, improper lifestyles, epigenetic dysregulations, environmental toxicants etc. Which ultimately leads to functional impairment followed by disease development. Abbreviation- Pb: Lead; Cd: Cadmium; Hg: Mercury; DNMT: DNA methyltransferase).

A number of factors are responsible for gametes specific developmental impairments and associated in utero or postnatal abnormalities. Proper development and functionality of a gene and also efficiency of the traits under control are dependent on the governing (regulating) signaling pathways, autocrine and paracrine factors, surrounding environmental cues and nourishing elements (in the form of cytokines and growth factors). Shortcomings in any of the above said necessities may cause partial or permanent defects. Diagrammatical representation of factors affecting germ cell development and their functionality has been depicted in Figure 3. These can be categorized as follows:

### 4.1. Chromosomal Aberrations

Chromosomal aberrations correspond to insertion, deletion or translocation of some or the whole part (arms) of a chromosome. These abnormalities have been associated with the occurrence of impaired functioning of the various body parts and hence are the causatives of many complications including ovarian germ cell tumors (OGSTs). According to Matyakhina et al., all malignant human tumors occur due to chromosomal aberrations induced chromosomal instability [40]. These disturbances happen due to abnormal segregation of chromosomes at the time of cell division. While observing abnormal karyotypes from the patients with OGSTs, comparative genomic hybridization (CGH) and fluorescence in situ hybridization (FISH) techniques revealed that gains or loss from the chromosomal arms or some of the whole chromosomes resulted into dysgerminomas (DGs), endodermal sinus tumors (ESTs) and immature teratomas [41,42,43]. The gain and loss of different chromosome numbers such as loss of 1p, gain of 1q or whole chromosomal gains chromosome numbers including 3, 8, 14 and 21 have been observed in female patients with malignant OGCTs. Among OGCTs, dysgerminomas were associated with abnormalities in 12p chromosome [41]. While performing dual color fluorescence in situ hybridization (FISH), Paolo Cossu-Rocca and colleagues observed that aberrations in the 12p chromosome were associated with 81% of total dysgerminomas [43] and seminomas (testicular germ cell tumor). Chromosomal abnormalities have also been correlated with ovarian granulosa cell tumors [44,45]. Comparative genomic hybridization and FISH techniques revealed that gains of chromosomes 12 and 14 and losses of chromosomes 22 and X were causatives of ovarian granulosa cell tumors [44]. In a similar study, patients with stage 1 granulosa cell tumors were shown to have chromosomal abnormalities in the form of losses from 1p33 to p36, 16p13.1, 16q and 22q chromosomes whereas gains were observed in chromosomes including 7p15 to p21, 12 and 14 [45]. Among these chromosomes, losses from chromosome 22 and gains in chromosome 14 were more prevalent. However, none of the conditions exhibited alarming degree of metastasis. In general, due to low prevalence rate (comparatively less than testicular germ cell tumors) in comparison to other extra-gonadal tumors, not many reports are available. However, it is equally important to draw attention on the role of chromosomal aberrations in the occurrence of diseases especially OGCTs as they can be diagnosed and can be proved helpful in avoiding future complications. Therefore, techniques like karyotyping, CGH and FISH should also be given preferences so that anomalies against normal behavior can be diagnosed and eradicated in time.

### 4.2. Spontaneous Genetic and Epigenetic Regulations

Epigenetics plays an important role in regulating overall transformation and functional activity of germ cells which upon fertilization can transmit proper information to the next generation [46,47]. A series of gradual events including PGC specification, meiotic entry, epigenetic imprinting of PGCs, histone modification and X-chromosome inactivation, DNA methylation erasure and its resumption during gamete formation, are highly important during gametogenesis [48]. The actual reason behind epigenetic information erasure in germ cells is not known but it may be attributed to the restricted transmission of unwanted acquired epimutations during life [20]. Although, except ESCs and iPSCs (only few reports), other stem cell sources derived gamete-like cells could either failed to initiate meiosis, or failed to prove their functionality by producing healthy viable offsprings upon fertilization. This may be perhaps due to developmental issues related to improper epigenetic modifications [48]. During migration period, PGCs go through genome wide DNA demethylation, histone modification by means of downregulation of H3 lysine 9 dimethylation (H3K9me2), upregulation of H3 lysine 27 trimethylation (H3K27me3), imprinting erasure and reactivation of X chromosome [16,17]. By the end of E12.5, H3K9me3 and H3K64me3 are gradually lost from PGCs and all these events are vital for efficient differentiation into gametes [49,50].

### 4.3. External/Environmental Factors and Incorrect Lifestyle Associated Defects/Exposure to Toxicants In Utero

A healthy life with proper functionality of all the organs depends a lot on many factors including surrounding environment, lifestyle, routine activities (exercising, food and drinking habits), and opted occupation. All these factors have also been associated with the reproductive life of an individual especially during female pregnancies. Here below is the detailed description about these factors in relation to female reproductive health.

#### 4.3.1. Chemical Intoxicants

Chemical intoxication especially during cancer treatments have been associated with reproductive disorders in both males and females. Fetal exposure to cyclophosphamide was shown to induce testicular germ cell tumor (TGCT), gonadal toxicity and reduced spermatogenesis [51]. Offspring produced from cyclophosphamide exposed mice showed high incidence of TGCT and reduced testicular and epididymal sperm count whereas primordial follicle loss and increased follicular growth activation was observed in female offspring. Endocrine disrupting compounds (EDCs) represents another category of reproductive toxicants which produces deleterious effects on male and female gametes by interfering natural hormone activities and other associated developmental processes [52]. Women (at different reproductive life stages) exposed to environmental di(2-ethylhexyl) phthalate (DEHP) and its metabolites were shown to have impaired reproduction and reduced fertility. Moreover, presence of DEHP and its major metabolite mono(2-ethyhexyl) phthalate (MEHP) in the urine samples of females have been associated with reduced number of high-quality fertilizable oocytes, and even early pregnancy loss [53,54,55]. In a recent study, Jing-Cai Liu et al. demonstrated that increased PDE3A expression in oocytes as a result of DEHP exposure results into impaired follicle assembly [56]. It was further evidenced that decreased levels of cAMP and PKA in oocytes resulted in an increase in PDE3A and ultimately caused impaired follicle assembly in DEHP-exposed ovaries. These compounds have also been demonstrated to cause deleterious reproductive effects, impaired folliculogenesis and ovulation, and decreased pregnancy rates in non-human species including rats, mice and cows [57,58,59]. Therefore, exposure to such deleterious and toxic chemicals must be avoided and in case of cancer treatment it is better to preserve the functional gametes so that fertility can be restored later on by transplanting back the autologous gametes into the donor body. Use of stem cells based therapeutic agents (stem cell therapy) or very small embryonic cell-like cells (which can survive cancer therapies) can be another efficient and safer way to restore fertility [4].

#### 4.3.2. Smoking, Alcoholism and Drug Abuse

Incorrect lifestyle, i.e., smoking, alcohol intake, and illicit drug consumption, also causes serious detrimental effects on reproductive lives of both males and females. The harmful effects of smoking on female reproductive system specifically impaired ovarian function were evidenced by decreased estradiol and progesterone production by in vitro cultured human luteinized granulosa cells under direct environmental tobacco smoke exposure [60]. A concentration (environmental tobacco smoke exposure) dependent increase in the expression of CYP1B1 both at gene and protein levels was seen in the cultured granulosa cells. High activity polymorphism of *CYP1B1* in cigarette smokers was also correlated with high risk of ovarian cancer development with disturbed hormonal regulation [61]. While analyzing retrieved ovarian cells from women undergoing assisted reproductive technologies (ART), major ovotoxic action of smoking was found to be oxidative stress and DNA damage in granulosa and oocyte cumulus cells, causing impaired functionality including improper oocyte maturation, gonadotropins binding to their receptors, increased zona pellucida thickness and reduced fertilization capacity [62,63,64,65]. In a cigarette smoke induced chronic obstructive pulmonary disease mouse model study, direct nasal exposure resulted into ovotoxicity by means of higher levels of primordial follicle depletion, apoptosis in antral follicle oocytes, induced oxidative stress and reduced availability of follicles for ovulation [66]. Similar ill effects were observed in other murine model studies targeting cigarette smoke induced ovarian impairment [67,68,69,70]. The effect of alcohol consumption on the female reproductive system and fertility has shown many contradictory results. In a study involving 124 patients undergoing routine urine inspection regarding steroidal hormone measurement, women with alcoholic habits during menstruation were shown to have >50% reduced conception probability during menstrual cycles [71]. Caffeine consumption further enhanced alcohol induced negative effects whereas no adverse effect was seen in participants consuming caffeine only. It has been reported that high consumption rate (rather than moderate alcohol consumption) results into decreased fertility [72]. However, a number of cohort case studies have exhibited no correlation between alcohol consumption and female infertility [73,74,75]. Illicit drug use has also been associated with the female reproductive system related issues. Previously, it was demonstrated that women with regular marijuana intake have increased risk for ovulatory dysfunction [76]. Another study concerning women with occasional or frequent marijuana intake habits, showed longer follicular phase, delayed ovulation, and slightly increased rate of ovulatory inhibition in comparison to non-marijuana users [77]. Contrastingly, in a recent report it was found that women with habits of co-use of marijuana with tobacco were shown to have shorter luteal phase than only tobacco users [78] however no differences between follicular phase and menstrual cycle lengths were observed among both types of participants. Missing correlating link between these studies corresponds to a number of factors including heterogeneity in the study design, volume or amount of cigarette/alcohol/illicit drug consumption on occasional or regular basis, exact time or duration of consumption (such as early age, during pregnancy or later stages), past intake history with no current consumption etc. Therefore, it’s hard to conclude whether such incorrect habits or life styles plays a direct or definite role in deteriorating reproductive life to a greater extent or not. However, as all of these lifestyles poses negative effects on the health therefore must be avoided much longer before conceiving or attaining pregnancy. Factors causing defects in germ cell development and fertility impairment has been depicted in Figure 3.

## 5. Germ Cell Tumor Development and Their Molecular Regulation

Female ovaries are the highly specialized organs which acts as reservoirs of highly potent cells with the ability to produce whole new life when fertilized with opposite sex gametes. However, adversities like old age, non-functionality due to developmental defects, and issues including tumor or cancer development are the unwanted threats which may cause infertility. Female germ cell tumors may be derived from various types of cells including surface epithelium, stroma, or follicular cell elements. Ovarian germ cell tumors (OGCTs) are mostly associated with early developmental stages of primitive germ cells of the embryonic gonad and are developed during specification, migration, and colonization of the primordial germ cells in the genital ridges [79,80].

### 5.1. Occurrence and Molecular Regulation

During PGC specification and germ cell development, *PRDM14* is required for early germ cell specification in mice [29]. Genome-wide association studies revealed that single nucleotide polymorphisms near the *PRDM14* gene have been associated with GCT development in mice [81]. Under normal conditions, PGCs undergo demethylation patterning during their migration towards gonads. Upon arrival at genital ridges, PGCs lose their migratory ability and resume their DNA methylation and transformed into male and female sex gametes. Any aberration in the migratory PGCs which are quite unstable due to their hypo methylated state, are eliminated by apoptosis. However, surviving aberrated PGCs develop into GCTs [25,82,83,84]. OGCTs arises as rare and potentially curable pathological conditions which constitutes about 2–3 percent of all ovarian cancers and have also been shown to affect females at their adolescence and median age [84]. In a recent review of literature, Boussios et al. has briefly explained about the role of somatic mutations and copy number variations in the resemblance of OGCTS with TGCTs and also discussed about how these mutations help specific disease conditions (i.e., tumor development) to attain resistance against chemical treatments [85]. They also advocated the necessity of unraveling immune checkpoint inhibitors. Therefore, such conditions must be given due importance when deciding therapeutical applications. Chromosomal abnormalities as a result of gain or loss of a part of the arm of chromosomes have been resulted in to a variety of OGCTs [40,41,42,43,44,45]. Their diagnoses have been made possible with the help of techniques like karyotyping, CGH and FISH. Depending upon various internal and external factors, ovarian surface epithelium (OSE) derived cells which surrounds ovary as a whitish layer have been found responsible for development of either neo-oogenesis or ovarian cancer [86]. Studies indicate that OSE associated cancers contributes to almost 90% of the total ovarian cancers [87]. In light of these cancer generating possibilities, researchers believed that removal of OSE can reduce the risk [88,89]. To check this hypothesis, Wright and colleagues used rhesus monkey model and found normal processes like menstruation, gonadotropin levels, and follicular development. Interestingly, no adverse effects were seen even after 6 and 12 month periods of OSE removal in the rhesus monkey models. However, long term effects on future ovarian cancer development were not evaluated as study was performed for only one year. Epithelial to mesenchymal transition (EMT) was found to be another factor associated with ovarian cancer development. While analyzing ovarian tissue sections of women with ovarian carcinoma using immunohistochemistry targeting pluripotency markers and Vimentin, small number of Vimentin and NANOG-positive cells with larger nuclei (release from surface epithelium) were shown to form cell clusters with altered mesenchyme like phenotype, and termed as cancer stem cells [90]. These cancer-like stem cells were thought to undergo EMT as they originated from OSE. This shift from epithelial to mesenchymal status was responsible for ovarian carcinoma progression under the effect of multiple genes mutations and signaling pathways which further resulted into downregulation of epithelial markers and upregulation of mesenchymal markers [91,92,93]. Therefore, markers, such as OCT3/4, NANOG, and Vimentin, can be used as diagnostic markers for ovarian cancer cell detection.

Immunohistochemistry has been the long and reliable tool for diagnosing OGCTs. However, proper fixation of the testing sample constitutes one of the critical parameter as poor fixation hampers the cell quality and also the results [94]. In many laboratories, diagnosis of dysgerminomas relies on the use of placental alkaline phosphatase (PLAP) staining but for poorly fixed materials it is unreliable. Use of podoplanin (D2-40) can be one of the reliable alternatives to this problem as it can be specifically used as a relatively stable marker because it can strongly stain both the cytoplasm and cell membranes of even poorly fixed or necrotic materials [95]. Protein expression of pluripotency associated markers has been shown by malignant conditions. While studying malignant GCTs, pluripotency marker LIN28A was shown to maintain undifferentiated state by repressing tumor suppressive let-7 miRNA family members [96], suggesting its use as a diagnostic marker for such complications. Similarly, epithelial cell adhesion molecule (EPCAM) has also been observed as a serum diagnostic marker for treating GCTs [97]. Improper pluripotency regulation during PGC specification and migration has also been suggested to be one of the reasons for type-1 GCTs i.e., teratomas and yolk sac tumors [86]. Specified PGCs under the control of regulating genes, such as *BLIMP1*, *PRDM14*, *STELLA*, and *FIGLA*, follow a definite migration route until their entry into gonadal ridges. Deviated migration leads to ectopic germ cell localization and constitutes risk for GCT development. Factors such as KITLG, CXCR4 and CXCR12 have also been associated with the regulation of PGC migration until gonadal ridges, knocking down of such important factors have resulted into migration failure and ultimately GCT development [98,99].

### 5.2. Types, Symptoms and Diagnosis

On the basis of occurrence and pathological conditions, GCTs have been classified into following categories, dysgerminomas, teratomas (immature and mature), endodermal sinus tumor or yolk sac tumors, choriocarcinomas, mixed germ cell tumors and embryonal carcinomas. Ovarian germ cell tumors are rare, typically manifested mostly in adolescence with symptoms like abdominal pain, vaginal bleeding, palpable mass development, diagnosed with elevated levels of tumor markers including alpha fetoprotein (AFP) and human chorionic gonadotrophin (hCG) and elevated levels of lactic dehydrogenase (LDH) and alkaline phosphatase (AP) in the serum [100,101]. Dysgerminomas which constitutes the most common GCT among all ovarian malignancies, are most commonly seen during adolescence or early childhood (sometimes in old age women too) are characterized as solid lobulated mass, having round PGC like cells with large flattened round nucleus. Dysgerminomas can be diagnosed by analyzing serum LDH, β-hCG and AP levels. Teratomas constitutes second most common form of GCTs after dysgerminomas [100,102]. They are further classified as mature and immature teratoma types. Mature teratomas are usually non-cancerous benign in nature however immature teratomas can generally develop into malignant cancers. Mature teratomas can be further divided into cystic, solid and mixed mature teratomas. Ovarian teratomas (mature and immature) are comprised of tissues derived from all the three germ layers and are seen in younger patients. Immature teratomas are usually larger in size than mature teratomas [100]. Ovarian teratomas mostly share symptoms like dysgerminomas and can be characterized as abdominal pain due to ovarian torsion, bleeding, and can be diagnosed with mild elevated levels of tumor markers AFP and hCG. Yolk sac tumors (YST) also known as endodermal sinus tumors constitutes the third commonest type of OGCTs after dysgerminomas and teratomas. Yolk sac tumors are large encapsulated tumors with smooth external surfaces, having primitive yolk sac like cellular structure and are found mostly in women with 20–30 years of age. However, few reports have also been seen in old age post-menopausal patients and their low occurrence may be attributed to the factors including poor diagnosis associated limited knowledge and scarce treatment outcomes [103]. Non regular AFP testing in postmenopausal patients and negligence about accurate histological identifications may be one of the reasons behind this issue. Presence of Schiller-Duval bodies has been considered as most diagnostic feature in most YSTs [101]. YSTs are diagnosed with tumor markers AFP and LDH however they may rarely show α1-antitrypsin. YSTs are OCT4 and CD30 negative in nature while shows positive expression for SALL4, keratins and glycan-3 [104,105,106]. Choriocarcinomas represents the rarest form of gonadal GCTs which occur due to cancerous transformation of the placental cells. Development of mono-nucleated syncytiotrophoblast and cytotrophoblast cells without formational of placental type villi constitute the characteristic feature of choriocarcinomas. Symptoms include the vaginal blessing, abdominal pain, cough, and headache. Hemoptysis (coughing up blood) has been considered as one of the first symptoms in the diagnosis of metastatic choriocarcinomas [107]. Choriocarcinomas are diagnosed with elevated levels of β-hCG and lump formation in the pelvic region. Ovarian mixed germ cell tumors represent mixing of more than one GCT such as dysgerminomas, YSTs, teratoma, etc. [100,101]. Symptoms are based on the type of tumor present in the patients. Like other GCTs, mixed germ cell tumors may be diagnosed with high serum levels of β-hCG, AFP or LDH. Embryonal carcinomas also represent the rare form of GCTs which comes under the mixed germ cell tumors category. Embryonal carcinomas show positive expression for OCT4, SALL4, CD30, glycan-3 and keratins [105]. They also show elevated levels of serum tumor markers such as AFP and β-hCG. Description about different types of tumors and associated diagnostic markers has been given in Table 1.

### 5.3. Treatment and Associated Complications

With the advancement in science and technology, a number of malignancies can be diagnosed and treated so that death rates associated with such complications can be minimized. On the basis of type, site of occurrence, and severity, a number of strategies involving surgery (unilateral or bilateral salpingo-oophorectomy, tumor debulking), adjuvant therapy, radiation therapy, chemotherapy (systemic chemotherapy, regional chemotherapy, combination chemotherapy), and high dose chemotherapy followed by bone marrow transplant or autologous stem cell infusion are frequently used. However, complications like tumor recurrence and side defects as a result of chemical or radiation-based therapies sometimes cause serious life threats to the patients. Under tumor re-occurrence circumstances, cumulative dose of etoposide must be considered during determination of number of chemotherapy delivery cycles to the patients [108]. However, associated side defects must be taken into account while using it. Usually patients with stage-1 OGCTs can be cured with surgery only and do not need adjuvant therapy [109,110], however, in advanced stage cases both surgery and adjuvant therapy are needed. In some cases, patients with advanced tumor stages develop resistance to certain chemicals namely cisplatin and need to be cured by combination chemotherapy. A number of factors are involved in GCT response to chemotherapy among which variable efficiency of the DNA damage response activation constitutes a major target. Associated factors include excision repair cross-complementation group 1 (ERCC1) expression [111], checkpoint kinase 2 (CHEK2) activation [112], and homologous recombination repair pathways activation [113,114]. These factors can be efficiently used in evaluation of GCT susceptibility in response to chemotherapeutic agents. As overall prevalence of GCTs is more in males, comparatively less studies related with resistance development in females have been reported. A number of factors including inactivation, elevation and downregulation of *p53*, *p21*, *MDM2*, and *Oct-4* and *Noxa* genes have been associated with cisplatin resistance [115,116,117]. Females who attained cisplatin resistance have been treated with combination therapies, such as cisplatin, vinblastine, bleomycin, or cyclophosphamide, vincristine, actinomycin D [118,119]. Patients undergone cisplatin resistance have also been administered with high dose chemotherapy along with peripheral blood progenitor cell infusion [120]. Use of chimeric antigen receptors (CAR) T-cell therapy must also be considered as it has been successfully administered in treating some of the chemo- resistant cancers [121]. In any of conditions, fertility restoration should be given utmost priority as most of the chemical and radiation therapies causes severe fertility problems.

## 6. Present Experimental Needs and Future Expectations

Science has been advanced to a greater extent with the development of many valuable techniques and associated improved methodologies. Among others, the use of stem cells as therapeutic agents have further revolutionized the scientific field especially where disease recovery and functional enhancement of any cell, tissue or organ is concerned. Utilization of bioengineered cells have made it possible to treat many pathological conditions in a much safer and effective way. Therefore, all such experiences can be used as a base to develop more novel strategies to eliminate yet unresolved issues not limited to female fertility but to other biological fields also. Fixed number of oocytes, age dependency, post chemotherapeutical threats, xenogeneic contamination problems (arose due to use of certain animal-based products during experimentation), functional incompetence (infertility), etc., constitutes major obstacles to this field. As far as stem cells based research is concerned, dependency on the use of ESCs and iPSCs to derive desirable results (due to the pluripotent nature) and related ethical and teratogenic concerns, use of follicular fluid derived from different organisms (porcine, bovine etc.), forced expression of targeted genes (transfection or ectopic expression), low potency of adult MSCs with reduced survivability during longer cultures and cryopreservation issues (reduced post thaw cell yield, cytotoxicity, and reduced stemness) constitutes the major obstacles. Keeping all these complications and necessities in mind, new experimental strategies need to be developed.

## 7. Conclusions

As germ cells have the capability to produce healthy offspring, their errorless development in appropriate way may hold greater possibilities for a healthy life for the present as well as future generations. Until maturity, germ cells undergo a series of events passing through various stages under different biological and environmental cues and any deformity or suppression of indispensable developmental factors either causes functional impairment or limits life expectancies. Therefore, it is highly important to understand the whole developmental process and all unnecessary and health hazardous things (improper lifestyle, heavy alcohol drinking, smoking, drug abuse, active and passive intoxication) should be avoided so that proper health can be maintained and delivered to future generations by means of healthy and functional gametes. To achieve this goal, more advanced studies will be needed so that yet unresolved mechanisms can be explored. Moreover, more focus has to be given to develop novel safer and highly efficient ways to restore the functionality of female germ cells which have limited numbers and age dependency. Additionally, stem cell-based therapies utilizing stem cells to develop (in vitro), cure and restore ovarian function can be of immense importance. Stem cell-based therapies can also be proven helpful to treat OGCTs with or without use of tumor destructive chemicals. However, to make all of these assumptions feasible still more extensive and valuable research outcomes are imperative.

## Figures and Tables

**Table 1:** Ovarian germ cell tumors: Types, prevalence, targeted age groups and diagnostic markers. Abbreviations: OGCTs: ovarian germ cell tumors; LDH: Lactic dehydrogenase; β-hCG: Beta-human chorionic gonadotrophin; AP: Alkaline phosphatase; AFP: Alpha fetoprotein.

Types	Prevalence Among OGCTs	Targeted Age Groups	Diagnostic Markers
Dysgerminomas	Most common type	Mostly occur in adolescence and early childhood, rarely old age	LDH, β-hCG, AP
Teratomas	Second most common type	Mostly occur during young age, rarely old age	AFP, β-hCG
Yolk-sac tumors	Third most common type	Mostly occur in women with 20–30 years age, rarely old age	Schiller Duval bodies, AFP, LDH, α1-antitrypsin
Choriocarcinomas	Rare form	During or after pregnancy	AFP, β-hCG
Mixed germ cell tumors	Common form	Different age groups	LDH, AFP, β-hCG
Embryonal carcinomas	Rare form	Predominantly occurs in children and adolescents	LDH, AFP, β-hCG

## Data Availability

Not applicable.

## References

[1] Hayashi M., Kawaguchi T., Durcova-Hills G., Imai H. (2018). Generation of germ cells from pluripotent stem cells in mammals. Reprod. Med. Biol..

[2] Felici M.D. (2010). Germ stem cells in the mammalian adult ovary: Considerations by a fan of the primordial germ cells. Mol. Hum. Reprod..

[3] Mochizuki K., Hayashi Y., Sekinaka T., Otsuka K., Matsuoka Y., Kobayashi H., Oki S., Takehara A., Kono T., Osumi N. (2018). Repression of Somatic Genes by Selective Recruitment of *HDAC3* by *BLIMP1* Is Essential for Mouse Primordial Germ Cell Fate Determination. Cell. Rep..

[4] Bharti D., Jang S.J., Lee S.Y., Lee S.L., Rho G.J. (2020). In Vitro Generation of Oocyte Like Cells and Their In Vivo Efficacy: How Far We have been Succeeded. Cells.

[5] Aramaki S., Hayashi K., Kurimoto K., Ohta H., Yabuta Y., Iwanari H., Mochizuki Y., Hamakubo T., Kato Y., Shirahige K. (2013). A mesodermal factor, *T*, specifies mouse germ cell fate by directly activating germline determinants. Dev. Cell.

[6] Ying Y., Qi X., Zhao G.Q. (2001). Induction of primordial germ cells from murine epiblasts by synergistic action of BMP4 and BMP8B signaling pathways. Proc. Natl. Acad. Sci. USA.

[7] Ying Y., Zhao G.Q. (2001). Cooperation of Endoderm-Derived BMP2 and Extraembryonic Ectoderm-Derived BMP4 in Primordial Germ Cell Generation in the Mouse. Dev. Biol..

[8] Lawson K.A., Dunn N.R., Roelen B.A., Zeinstra L.M., Davis A.M., Wright C.V., Korving J.P., Hogan B.L.M. (1999). Bmp4 is required for the generation of primordial germ cells in the mouse embryos. Genes. Dev..

[9] Tremblay K.D., Dunn N.R., Robertson E.J. (2001). Mouse embryos lacking *Smad1* signals display defects in extra-embryonic tissues and germ cell formation. Development.

[10] Chu G.C., Dunn N.R., Anderson D.C., Oxburgh L., Robertson E.J. (2004). Differential requirements for *Smad4* in TGFβ-dependent patterning of the early mouse embryo. Development.

[11] Chang H., Matzuk M.M. (2001). *Smad5* is required for mouse primordial germ cell development. Mech. Dev..

[12] Cantú A.V., Laird D.J. (2017). Primordial germ cell migration and the Wnt signaling pathway. Anim. Reprod..

[13] Saitou M., Payer B., Lange U.C., Erhardt S., Barton S.C., Surani M.A. (2003). Specification of germ cell fate in mice. Philos. Trans. R. Soc. Lond. B Biol. Sci..

[14] Lange U.C., Saitou M., Western P.S., Barton S.C., Surani M.A. (2003). The Fragilis interferon-inducible gene family of transmembrane proteins is associated with germ cell specification in mice. BMC. Dev. Biol..

[15] Tres L.L., Rosselot C., Kierszenbaum A.L. (2004). Primordial Germ Cells What Does It Take to be alive?. Mol. Reprod. Dev..

[16] Saitou M., Kagiwada S., Kurimoto K. (2012). Epigenetic reprogramming in mouse pre-implantation development and primordial germ cells. Development.

[17] Lee H.J., Hore T.A., Reik W. (2014). Reprogramming the Methylome: Erasing Memory and Creating Diversity. Cell Stem Cell.

[18] Gkountela S., Zhang K.X., Shafiq T.A., Liao W.W., Calvopiña J.H., Chen P.Y., Clark A.T. (2015). DNA Demethylation Dynamics in the Human Prenatal Germline. Cell.

[19] Guo F., Yan L., Guo H., Li L., Hu B., Zhao Y., Yong J., Hu Y., Wang X., Wei Y. (2015). The Transcriptome and DNA Methylome Landscapes of Human Primordial Germ Cells. Cell.

[20] Canovas S., Campos R., Aguilar E., Cibelli J.B. (2017). Progress towards human primordial germ cell specification in vitro. Mol. Hum. Reprod..

[21] Chen D., Sun N., Hou L., Kim R., Faith J., Aslanyan M., Tao Y., Zheng Y., Fu J., Liu W. (2019). Human Primordial Germ Cells Are Specified from Lineage-Primed Progenitors. Cell Rep..

[22] Chen D., Liu W., Lukianchikov A., Hancock G.V., Zimmerman J., Lowe M.G., Kim R., Galic Z., Irie N., Surani M.A. (2017). Germline competency of human embryonic stem cells depends on eomesodermin. Biol. Reprod..

[23] Irie N., Weinberger L., Tang W.W.C., Kobayashi T., Viukov S., Manor Y.S., Dietmann S., Hanna J.H., Surani M.A. (2015). SOX17 is a critical specifier of human primordial germ cell fate. Cell.

[24] Sasaki K., Yokobayashi S., Nakamura T., Okamoto I., Yabuta Y., Kurimoto K., Ohta H., Moritoki Y., Iwatani C., Tsuchiya H. (2015). Robust In Vitro Induction of Human Germ Cell Fate from Pluripotent Stem Cells. Cell Stem Cell.

[25] Raz E. (2003). Primordial germ-cell development: The zebrafish perspective. Nat. Rev. Genet..

[26] Richardson B.E., Lehmann R. (2010). Mechanisms guiding primordial germ cell migration: Strategies from different organisms. Nat. Rev. Mol. Cell Biol..

[27] Saitou M., Payer B., O’Carroll D., Ohinata Y., Surani M.A. (2005). *Blimp1* and the emergence of the germ line during development in the mouse. Cell Cycle.

[28] Vincent S.D., Dunn N.R., Sciammas R., Sharpiro-Shalef M., Davis M.M., Calame K., Bikoff E.K., Robertson E.J. (2005). The zinc finger transcriptional repressor *Blimp1/Prdm1* is dispensable for early axis formation but is required for specification of primordial germ cells in the mouse. Development.

[29] Kurimoto K., Yamaji M., Seki Y., Saitou M. (2008). Specification of the germ cell lineage in mice: A process orchestrated by the PR-domain proteins, Blimp1 and Prdm14. Cell Cycle.

[30] Yamaji M., Seki Y., Kurimoto K., Yabuta Y., Yuasa M., Shigeta M., Yamanaka K., Ohinata Y., Saitou M. (2008). Critical function of Prdm14 for the establishment of the germ cell lineage in mice. Nat. Genet..

[31] Tanaka S.S., Yamaguchi Y.L., Tsoi B., Lickert H., Tam P.P. (2005). IFITM/Mil/fragilis family proteins IFITM1 and IFITM3 play distinct roles in mouse primordial germ cell homing and repulsion. Dev. Cell.

[32] Lange U.C., Adams D.J., Lee C., Barton S., Schneider R., Bradley A., Surani M.A. (2008). Normal germ line establishment in mice carrying a deletion of the *Ifitm/Fragilis* gene family cluster. Mol. Cell Biol..

[33] Weber S., Eckert D., Nettersheim D., Gillis A.J., Schafer S., Kuckenberg P., Ehlermann J., Werling U., Biermann K., Looijenga L.H. (2010). Critical function of AP-2g/TCFAP2C in mouse embryonic germ cell maintenance. Biol. Reprod..

[34] Julaton V.T.A., Pera R.A.R. (2011). *NANOS3* function in human germ cell development. Hum. Mol. Genet..

[35] Shen W., Zhang D., Qing T., Cheng J., Bai Z., Shi Y., Ding M., Deng H. (2006). Live Offspring Produced by Mouse Oocytes Derived from Premeiotic Fetal Germ Cells. Biol. Reprod..

[36] Zou K., Yuan Z., Yang Z., Luo H., Sun K., Zhou L., Xiang J., Shi L., Yu Q., Zhang Y. (2009). Production of offspring from a germline stem cell line derived from neonatal ovaries. Nat. Cell Biol..

[37] Hayashi K., Ogushi S., Kurimoto K., Shimamoto S., Ohta H., Saitou M. (2012). Offspring from Oocytes Derived from in Vitro Primordial Germ Cell–like Cells in Mice. Science.

[38] Morohaku K., Tanimoto R., Sasaki K., Kawahara-Miki R., Kono T., Hayashi K., Hiraoe Y., Obata Y. (2016). Complete in vitro generation of fertile oocytes from mouse primordial germ cells. Proc. Natl. Acad. Sci. USA.

[39] Tian C., Liu L., Ye X., Fu H., Sheng X., Wang L., Wang H., Heng D., Liu L. (2019). Functional Oocytes Derived from Granulosa Cells. Cell Rep..

[40] Matyakhina L., Lenherr S.M., Stratakis C.A. (2002). Protein Kinase A and Chromosomal Stability. Ann. N. Y. Acad. Sci..

[41] Kraggerud S.M., Szymanska J., Abeler V.M., Kaern J., Eknaes M., Heim S., Teixeira M.R., Trope C.G., Peltomäki P., Lothe R.A. (2000). DNA Copy Number Changes in Malignant Ovarian Germ Cell Tumors. Cancer Res..

[42] Bussey K.J., Lawce H.J., Himoe E., Shu X.O., Suijkerbuijk R.F., Olson S.B., Magenis R.E. (2001). Chromosomes 1 and 12 abnormalities in pediatric germ cell tumors by interphase fluorescence in situ hybridization. Cancer Genet. Cytogenet..

[43] Cossu-Rocca P., Zhang S., Roth L.M., Eble J.N., Zheng W., Abdul Karim F.W., Michael H., Emerson R.E., Jones T.D., Hattab E.M. (2006). Chromosome 12p abnormalities in dysgerminoma of the ovary: A FISH analysis. Mod. Pathol..

[44] Mayr D., Kaltz-Wittmer C., Arbogast S., Amann G., Aust D.E., Diebold J. (2002). Characteristic Pattern of Genetic Aberrations in Ovarian Granulosa Cell Tumors. Mod. Pathol..

[45] Lin Y.S., Eng H.L., Jan Y.J., Lee H.S., Ho W.L., Liou C.P., Lee W.Y., Tzeng C.C. (2005). Molecular cytogenetics of ovarian granulosa cell tumors by comparative genomic hybridization. Gynecol. Oncol..

[46] Feng L., Chen X. (2015). Epigenetic Regulation of Germ Cells— Remember or Forget?. Curr. Opin. Genet. Dev..

[47] Cinalli R.M., Rangan P., Lehmann R. (2008). Germ cells are forever. Cell.

[48] Sun Y.C., Wang Y.Y., Ge W., Cheng S.F., Dyce P.W., Shen W. (2017). Epigenetic regulation during the differentiation of stem cells to germ cells. Oncotarget.

[49] Hajkova P., Ancelin K., Waldmann T., Lacoste N., Lange U.C., Cesari F., Lee C., Almouzni G., Schneider R., Surani M.A. (2008). Chromatin dynamics during epigenetic reprogramming in the mouse germ line. Nature.

[50] Daujat S., Weiss T., Mohn F., Lange U.C., Ziegler-Birling C., Zeissler U., Lappe M., Schübeler D., Torres-Padilla M.E., Schneider R. (2009). H3K64 trimethylation marks heterochromatin and is dynamically remodeled during developmental reprogramming. Nat. Struct. Mol. Biol..

[51] Paul B.C., Ana L.D., Hazel L.K., Richard A.A., Angabin M., Marvin L.M., Gunapala S. (2014). Fetal Cyclophosphamide Exposure Induces Testicular Cancer and Reduced Spermatogenesis and Ovarian Follicle Numbers in Mice. PLoS ONE.

[52] Zvi R., Alisa K.E., Dorit K. (2020). Effect of environmental contamination on female and male gametes—A lesson from bovines. Anim. Reprod..

[53] Toft G., Jönsson B.A.G., Lindh C.H., Jensen T.K., Hjollund N.H., Vested A., Bonde J.P. (2012). Association between Pregnancy Loss and Urinary Phthalate Levels around the Time of Conception. Environ. Health Perspect..

[54] Tranfo G., Caporossi L., Paci E., Aragona C., Romanzi D., De Carolis C., De Rosa M., Capanna S., Papaleo B., Pera A. (2012). Urinary phthalate monoesters concentration in couples with infertility problems. Toxicol. Lett..

[55] Machtinger R., Gaskins A.J., Racowsky C., Mansur A., Adir M., Baccarelli A.A., Calafat A.M., Hauser R. (2018). Urinary concentrations of biomarkers of phthalates and phthalate alternatives and IVF outcomes. Environ. Int..

[56] Liu J.C., Yan Z.H., Li B., Yan H.C., Felici M.D., Shen D. (2020). Di (2-ethylhexyl) phthalate impairs primordial follicle assembly by increasing PDE3A expression in oocytes. Environ. Pollut..

[57] Gupta R.K., Singh J.M., Leslie T.C., Meachum S., Flaws J.A., Yao H.H. (2010). C-(2-ethylhexyl) phthalate and mono-(2-ethylhexyl) phthalate inhibit growth and reduce estradiol levels of antral follicles in vitro. Toxicol. Appl. Pharmacol..

[58] Hannon P.R., Niermann S., Flaws J.A. (2016). Acute exposure to Di(2-Ethylhexyl) phthalate in adulthood causes adverse reproductive outcomes later in life and accelerates reproductive aging in female mice. Toxicol. Sci..

[59] Kalo D., Roth Z. (2017). Low level of mono(2-ethylhexyl) phthalate reduces oocyte developmental competence in association with impaired gene expression. Toxicology.

[60] Justin D.V., Catherine A.V., Craig B.M., Nicolas R.L., Alan J.C. (2006). In vitro exposure to environmental tobacco smoke induces CYP1B1 expression in human luteinized granulosa cells. Reprod. Toxicol..

[61] Goodman M.T., McDuffie K., Kolonel L.N., Terada K., Donlon T.A., Wilkens L.R., Guo C., Marchand L.L. (2001). Case-control study of ovarian cancer and polymorphisms in genes involved in catecholestrogen formation and metabolism. Cancer Epidemiol. Biomark. Prev..

[62] Alviggi C., Cariati F., Conforti A., De Rosa P., Vallone R.S.I., Pivonello R., De Placido G. (2016). The effect of FT500 Plus^®^ on ovarian stimulation in PCOS women. Reprod. Toxicol..

[63] Budani M.C., Carletti E., Tiboni G.M. (2017). Cigarette smoke is associated with altered expression of antioxidant enzymes in granulosa cells from women undergoing in vitro fertilization. Zygote.

[64] Sinko I., Morocz M., Zadori J., Kokavszky K., Rasko I. (2005). Effect of cigarette smoking on DNA damage of human cumulus cells analyzed by comet assay. Reprod. Toxicol..

[65] Shiloh H., Lahav-Baratz S., Koifman M., Ishai D., Bidder D., Weiner-Meganzi Z., Dirnfeld M. (2004). The impact of cigarette smoking on zona pellucida thickness of oocytes and embryos prior to transfer into the uterine cavity. Hum. Reprod..

[66] Sobinoff A.P., Beckett E.L., Jarnicki A.G., Sutherland J.M., McCluskey A., Hansbro P.M., McLaughlin E.A. (2013). Scrambled and fried: Cigarette smoke exposure causes antral follicle destruction and oocyte dysfunction through oxidative stress. Toxicol. Appl. Pharmacol..

[67] Jennings P.C., Merriman J.A., Beckett E.L., Hansbro P.M., Jones K.T. (2011). Increased zona pellucida thickness and meiotic spindle disruption in oocytes from cigarette smoking mice. Hum. Reprod..

[68] Paixao L.L., Gaspar-Reis R.P., Gonzalez G.P., Santos A.S., Santana A.C., Santos R.M., Spritzer P.M., Nascimento-Saba C.C. (2012). Cigarette smoke impairs granulosa cell proliferation and oocyte growth after exposure cessation in young Swiss mice: An experimental study. J. Ovarian Res..

[69] Mai Z., Lei M., Yu B., Du H., Liu J. (2014). The effects of cigarette smoke extract on ovulation, oocyte morphology and ovarian gene expression in mice. PLoS ONE.

[70] Lee H.M., Kim C.W., Hwang K.A., Sung J.H., Lee J.K., Choi K.C. (2017). Cigarette smoke impaired maturation of ovarian follicles and normal growth of uterus inner wall of female wild-type and hypertensive rats. Reprod. Toxicol..

[71] Hakim R.B., Gray R.H., Zakur H. (1998). Alcohol and caffeine consumption and decreased fertility. Fertil. Steril..

[72] Eggert J., Theobald H., Engfeldt H. (2004). Effects of alcohol consumption on female fertility during an 18-year period. Fertil. Steril..

[73] Juhl M., Andersen A.M.N., Gronbaek M., Olsen J. (2001). Moderate alcohol consumption and waiting time to pregnancy. Hum. Reprod..

[74] Tolstrup J.S., Kjaer S.K., Holst C., Sharif H., Munk C., Osler M., Schmidt L., Andersen A.M.N., Gronbaek M. (2003). Alcohol use as predictor for infertility in representative population of Danish women. Acta Obstet. Gynecol. Scand..

[75] Chavarro J.E., Rich-Edwards J.W., Rosner B.A., Willett W.C. (2009). Caffeinated and Alcoholic Beverage Intake in Relation to Ovulatory Disorder Infertility. Epidemiology.

[76] Mueller B.A., Daling J.R., Weiss N.S., Moore D.E. (1990). Recreational drug use and the risk of primary infertility. Epidemiology.

[77] Jukic A.M.Z., Weinberg C.R., Baird D.D., Wilcox A.J. (2007). Lifestyle and reproductive factors associated with follicular phase length. J. Womens Health (Larchmt).

[78] Lammert S., Harrison K., Tosun N., Allen S. (2018). Menstrual Cycle in Women Who Co-use Marijuana and Tobacco. J. Addict. Med..

[79] Kraggerud S.M., Hoei-Hansen C.E., Alagaratnam S., Skotheim R.I., Abeler V.M., Meyts E.R.D., Lothe R.A. (2013). Molecular Characteristics of Malignant Ovarian Germ Cell Tumors and Comparison With Testicular Counterparts: Implications for Pathogenesis. Endocr. Rev..

[80] Muller M.R., Skowron M.A., Albers P., Nettersheim D. (2020). Molecular and epigenetic pathogenesis of germ cell tumors. Asian J. Urol..

[81] Ruark E., Seal S., McDonald H., Zhang F., Elliot A., Lau K.W., Perdeaux E., Rapley E., Eeles R., Peto J. (2013). Identification of nine new susceptibility loci for testicular cancer, including variants near *DAZL* and *PRDM14*. Nat. Genet..

[82] Kim S., Günesdogan U., Zylicz J.J., Hackett J.A., Cougot D., Bao S., Lee C., Dietmann S., Allen G.E., Sengupta R. (2014). PRMT5 protects genomic integrity during global DNA demethylation in primordial germ cells and preimplantation embryos. Mol. Cell.

[83] Lee A.S., Tang C., Rao M.S., Weissman I.L., Wu J.C. (2013). Tumorigenicity as a clinical hurdle for pluripotent stem cell therapies. Nat. Med..

[84] Pierce J.L., Frazier A.L., Amatruda J.F. (2018). Pediatric germ cell tumors: A developmental perspective. Adv. Urol..

[85] Boussios S., Mikropoulos C., Samartzis E., Karihtala P., Moschetta M., Sheriff M., Karathanasi A., Sadauskaite A., Rassy E., Pavlidis N. (2020). Wise Management of Ovarian Cancer: On the Cutting Edge. J. Pers. Med..

[86] Xu J., Zheng T., Hong W., Ye H., Hu C., Zheng Y. (2018). Mechanism for the Decision of Ovarian Surface Epithelial Stem Cells to Undergo Neo-Oogenesis or Ovarian Tumorigenesis. Cell Physiol. Biochem..

[87] Kurman R.J., Shih I.M. (2010). The origin and pathogenesis of epithelial ovarian cancer: A proposed unifying theory. Am. J. Surg. Pathol..

[88] Wright J.W., Pejovic T., Lawson M., Jurevic L., Hobbs T., Stouffer R.L. (2010). Ovulation in the Absence of the Ovarian Surface Epithelium in the Primate. Biol. Reprod..

[89] Wright J.W., Pejovic T., Jurevic L., Bishop C.V., Hobbs T., Stouffer R.L. (2011). Ovarian surface epitheliectomy in the non-human primate: Continued cyclic ovarian function and limited epithelial replacement. Hum. Reprod..

[90] Suster N.K., Smrkolj S., Virant-Klun I. (2017). Putative stem cells and epithelial-mesenchymal transition revealed in sections of ovarian tumor in patients with serous ovarian carcinoma using immunohistochemistry for Vimentin and pluripotency-related markers. J. Ovarian Res..

[91] Zhu Y., Nilsson M., Sundfeldt K. (2010). Phenotypic plasticity of the ovarian surface epithelium: TGF-beta 1 induction of epithelial to mesenchymal transition (EMT) in vitro. Endocrinology.

[92] Liu S., Sun J., Cai B., Xi X., Yang L., Zhang Z., Feng Y., Sun Y. (2016). NANOG regulates epithelial-mesenchymal transition and chemo-resistance through activation of the STAT3 pathway in epithelial ovarian cancer. Tumour Biol..

[93] Li X., Yang J., Wang X., Li X., Liang J., Xing H. (2016). Role of TWIST2, E-cadherin and Vimentin in epithelial ovarian carcinogenesis and prognosis and their interaction in cancer progression. Eur. J. Gynaecol. Oncol..

[94] Nogales F.F., Dulcey I., Preda O. (2014). Germ cell tumors of the ovary: An update. Arch. Pathol. Lab. Med..

[95] Chang M.C., Vargas S.O., Hornick J.L., Hirsch M.S., Crum C.P., Nucci M.R. (2009). Embryonic stem cell transcription factors and D2-40 (podoplanin) as diagnostic immunohistochemical markers in ovarian germ cell tumors. Int. J. Gynecol. Pathol..

[96] Murray M.J., Saini H.K., Siegler C.A., Hanning J.E., Barker E.M., Dongen S.V., Ward D.M., Raby K.L., Groves I.J., Scarpini C.G. (2013). LIN28 expression in malignant germ cell tumors downregulates let-7 and increases oncogene levels. Cancer Res..

[97] Schonberger S., Okpanyi V., Calaminus G., Heikaus S., Leuschner I., Nicholson J.C., Stoecklein N.H., Schneider D.T., Borkhardt A. (2013). EPCAM-A novel molecular target for the treatment of pediatric and adult germ cell tumors. Genes Chromosomes Cancer.

[98] Poynter J.N., Hooten A.J., Frazier A.L., Ross J.A. (2012). Associations between variants in KITLG, SPRY4, BAK1, and DMRT1 and pediatric germ cell tumors. Genes Chromosomes Cancer.

[99] Molyneaux K.A., Zinszner H., Kunwar P.S., Schaible K., Stebler J., Sunshine M.J., O’Brien W., Raz E., Littman D., Wylie C. (2003). The chemokine SDF1/CXCL12 and its receptor CXCR4 regulate mouse germ cell migration and survival. Development.

[100] Shaaban A.M., Rezvani M., Elsayes K.M., Baskin H., Mourad A., Foster B.R., Jarboe E.A., Menias C.O. (2014). Ovarian Malignant Germ Cell Tumors: Cellular Classification and Clinical and Imaging Features. Radiographics.

[101] Jamshidi P., Taxy J.B. (2020). Educational Case: Yolk Sac (Endodermal Sinus) Tumor of the Ovary. Acad. Pathol..

[102] Lakshmanan M., Gupta S., Kumar V., Akhtar N., Chaturvedi A., Misra S., Jain K., Garg S. (2018). Germ Cell Tumor Ovary: An Institutional Experience of Treatment and Survival Outcomes. Indian J. Surg. Oncol..

[103] Boussios S., Attygalle A., Hazell S., Moschetta M., Mclachlan J., Okines A., Banerjee S. (2015). Malignant Ovarian Germ Cell Tumors in Postmenopausal Patients: The Royal Marsden Experience and Literature Review. Anticancer Res..

[104] Hong R., Suh C.H., Lee M.J. (2007). Adenocarcinoma with Yolk Sac Tumor of the Stomach: Case Report with Review of the Literature and an Immunohistochemical Study. Korean J. Pathol..

[105] Cheng L., Zhang S., Talerman A., Roth L.M. (2010). Morphologic, immunohistochemical, and fluorescence in situ hybridization study of ovarian embryonal carcinoma with comparison to solid variant of yolk sac tumor and immature teratoma. Hum. Pathol..

[106] Wang F., Liu A., Peng Y., Rakheja D., Wei L., Xue D., Allan R.W., Molberg K.H., Li J., Cao D. (2009). Diagnostic utility of SALL4 in extragonadal yolk sac tumors: An immunohistochemical study of 59 cases with comparison to placental-like alkaline phosphatase, alpha-fetoprotein, and glypican-3. Am. J. Surg. Pathol..

[107] Álvarez-Sarrado L., González-Ballano I., Herrero-Serrano R., Giménez-Molina C., Rodríguez-Solanilla B., Campillos-Maza J.M. (2020). Hemoptysis as the first symptom in the diagnosis of metastatic choriocarcinoma in the third trimester of pregnancy: A case report. Case Rep. Womens Health.

[108] Boussios S., Zarkavelis G., Seraj1 E., Zerdes I., Tatsi K., Pentheroudakis G. (2016). Non-epithelial Ovarian Cancer: Elucidating Uncommon Gynaecological Malignancies. Anticancer Res..

[109] Gershenson D.M. (2012). Current advances in the management of malignant germ cell and sex cord-stromal tumors of the ovary. Gynecol. Oncol..

[110] Mangili G., Sigismondi C., Lorusso D., Cormio G., Candiani M., Scarfone G., Mascilini F., Gadducci A., Mosconi A.M., Scollo P. (2017). The role of staging and adjuvant chemotherapy in stage I malignant ovarian germ cell tumors (MOGTs): The MITO-9 study. Ann. Oncol..

[111] Azambuja A.A., Engroff P., Silva B.T., Zorzetti R.C.S., Morrone F.B. (2020). Evaluation of nuclear NF-κB, transglutaminase2, and ERCC1 as predictors of platinum resistance in testicular tumors. Int. Braz. J. Urol..

[112] AlDubayan S.H., Pyle L.C., Gamulin M., Kulis T., Moore N.D., Taylor-Weiner A., Hmaid A.A., Reardon B., Wubbenhorst B., Godse R. (2019). Association of Inherited Pathogenic Variants in Checkpoint Kinase 2 (CHEK2) With Susceptibility to Testicular Germ Cell Tumors. JAMA Oncol..

[113] Cavallo F., Feldman D.R., Barchi M. (2013). Revisiting DNA damage repair, p53-mediated apoptosis and cisplatin sensitivity in germ cell tumors. Int. J. Dev. Biol..

[114] Lobo J., Constancio V., Guimaraes-Teixeira C., Leite-Silva P., Miranda-Goncalves V., Sequeira J.P., Pistoni L., Guimaraes R., Cantante M., Braga I. (2021). Promoter methylation of DNA homologous recombination genes is predictive of the responsiveness to PARP inhibitor treatment in testicular germ cell tumors. Mol. Oncol..

[115] Koster R., Di Pietro A., Timmer-Bosscha H., Gibcus J.H., Van Den Berg A., Suurmeijer A.J., Bischoff R., Gietema J.A., De Jong S. (2010). Cytoplasmic p21 expression levels determine cisplatin resistance in human testicular cancer. J. Clin. Investig..

[116] Koster R., van Vugt M.A.T.M., Timmer-Bosscha H., Gietema J.A., de Jong S. (2013). Unravelling mechanisms of cisplatin sensitivity and resistance in testicular cancer. Expert Rev. Mol. Med..

[117] Gutekunst M., Mueller T., Weilbacher A., Dengler M.A., Bedke J., Kruck S., Oren M., Aulitzky W.E., Van Der Kuip H. (2013). Cisplatin hypersensitivity of testicular germ cell tumors is determined by high constitutive *Noxa* levels mediated by *Oct-4*. Cancer Res..

[118] Pawinski A., Favalli G., Ploch E., Sahmoud T., Van Oosterom A.T., Pecorelli S. (1998). PVB chemotherapy in patients with recurrent or advanced dysgerminoma: A phase II study of the EORTC Gynaecological Cancer Cooperative Group. Clin. Oncol..

[119] Ray-Coquard I., Morice P., Lorusso D., Prat J., Oaknin A., Pautier P., Colombo N., ESMO Guidelines Committee (2018). Non-epithelial ovarian cancer: ESMO Clinical Practice Guidelines for diagnosis, treatment and follow-up. Ann. Oncol..

[120] Wang J., Zhuo X., Yang J., Cao D., Shen K., Huang H., Wu M., Pan L., Xiang Y., Guo L. (2020). Outcomes and Prognostic Factors of Patients With Recurrent and Persistent Malignant Ovarian Germ Cell Tumors. Arch. Gynecol. Obstet..

[121] Uccello M., Boussios S., Samartzis E.P., Moschetta M. (2020). Systemic anti-cancer treatment in malignant ovarian germ cell tumours (MOGCTs): Current management and promising approaches. Ann. Transl. Med..

